# Multi-Objective Toughness Optimization of Epoxy Resin for Steel Bridge Deck Pavement Based on Crosslink Density Regulation

**DOI:** 10.3390/polym17101422

**Published:** 2025-05-21

**Authors:** Yixin Zhou, Gang Xu, Yulou Fan, Yuxiang Li, Xianhua Chen, Jun Yang, Wei Huang

**Affiliations:** 1 School of Transportation, Southeast University, Nanjing 210096, China101300349@seu.edu.cn (G.X.); yangjun@seu.edu.cn (J.Y.); 2 Intelligent Transportation System Research Center, Southeast University, Nanjing 210096, China

**Keywords:** steel bridge deck pavement, epoxy resin, toughness optimization, curing kinetics, response surface methodology

## Abstract

Epoxy resins (ERs) are esteemed for their mechanical robustness and adhesive qualities, particularly in steel bridge deck applications. Nonetheless, their intrinsic brittleness limits broader utility. This study addresses this limitation by modulating ER crosslink density through adjustments in curing agent concentration, incorporation of hyperbranched polymers (HBPs), and optimization of curing conditions. Employing a multi-objective optimization strategy, this research aims to enhance toughness while minimizing strength degradation. Non-isothermal curing kinetics, realized using the differential scanning calorimetry (DSC) method, attenuated total reflection Fourier transform infrared spectroscopy (ATR-FTIR), tensile testing, and thermogravimetric analysis (TGA), were employed to investigate the effects of curing agent and HBP content on the curing reaction, mechanical properties, and thermal stability, respectively. Response surface methodology facilitated comprehensive optimization. Findings indicate that both curing agent and HBP contents significantly influence curing dynamics and mechanical performance. Curing agent content below 40% or above 50% can induce side reactions, adversely affecting properties. While a curing agent content exceeding 45% or an HBP content exceeding 5% improves the toughness of ER, these increases concurrently reduce mechanical strength and thermal stability. The study identifies an optimal formulation comprising 45.21% curing agent, a curing temperature of 60.45 °C, and 5.77% HBP content.

## 1. Introduction

Epoxy resins are esteemed for their exceptional mechanical strength, chemical resistance, and adhesion properties, rendering them indispensable in pavement engineering [1,2,3,4]. The high rigidity and strength of epoxy networks lend themselves well to structural reinforcement [5,6]. However, these advantages are tempered by the inherent brittleness, restricting their performance in applications that require high flexibility or impact resistance [7,8]. Consequently, enhancing the toughness of epoxy resins has become a primary research focus to expand their functional versatility in demanding conditions.

To address this limitation, researchers have explored numerous modifiers to improve the toughness of epoxy resins, including rubber particles [9], core–shell polymer particles [10,11], nanoparticles [11,12], and hyperbranched polymers [13,14]. Wu et al. [15] investigated the critical particle size necessary for altering epoxy resins from brittle to ductile fracture behaviors, suggesting that rubber particles need to be below a specific critical size to achieve effective toughening. Quan et al. [11] incorporated nano-core–shell rubber (CSR) into a diglycidyl ether of bisphenol A (DGEBA) resin system, examining the effects of CSR particle size and volume fraction on toughening performance. The findings indicated that an effective CSR volume fraction of 30% maximized fracture energy improvements. Rajsekhar [12] further explored the influence of nano-Al_2_O_3_ on epoxy mechanical properties, observing that increased nano-Al_2_O_3_ content significantly enhanced fracture toughness and broadened the fracture process zone, demonstrating a substantial toughening effect. However, field emission scanning electron microscopy (FESEM) analysis revealed that excessive nano-Al_2_O_3_ concentrations could lead to particle agglomeration, potentially hindering uniform dispersion within the resin matrix.

A prominent strategy to improve the toughness of epoxy resins involves the integration of modifiers, such as rubber particles, nanomaterials, and other additives, which can induce shear banding or crazing formation, thereby dissipating energy during microcrack propagation. While this approach effectively decelerates crack growth, it does not fully inhibit crack formation, and the modifiers can often segregate or agglomerate within the epoxy resin matrix, reducing effectiveness. An alternative approach leverages modifiers with active functional groups (e.g., amino, carboxyl, hydroxyl, and isocyanate) capable of reacting with the epoxy groups or secondary hydroxyls within the resin. This interaction modifies the internal cross-linking network, thus reducing stiffness and enhancing toughness [16].

Hyperbranched polymers (HBPs) present a promising advancement in this area due to their unique, highly branched three-dimensional architecture, which enhances toughness while preserving material strength. The low viscosity of HBPs broadens the processing window of epoxy resins, positioning them as a significant focus in epoxy toughening research. Boogh et al. [17] examined dendritic polymers capped with epoxy groups to reinforce bisphenol F-type epoxy resins, observing that a 5 wt% addition of HBP increased the critical energy release rate by sixfold and fracture toughness (K_Ic_) by 2.5 times compared to unmodified resins, all while maintaining matrix strength, glass transition temperature, and processability. Cong et al. [14,18] further modified HBPs by converting hydroxyl terminal groups to epoxy end groups, which improved viscosity reduction, compatibility, and toughness. Zhang et al. [19] synthesized a novel hyperbranched flame-retardant polymer (ITA-HBP) from itaconic anhydride, achieving enhancements of 133.2% in impact strength, 78.7% in fracture toughness, and 124.7% in fracture energy in the modified epoxy resin. This modification also improved the resin’s flame-retardant performance, as reflected in an ultimate oxygen index of 36.3%.

Modifying the epoxy resin crosslink network is thus a highly effective approach, significantly improving toughness with minimal impact on strength, while simultaneously enhancing curing characteristics and processing flexibility. This method offers an optimal pathway for advancing epoxy resin performance in high-demand applications. According to reaction thermodynamics, increasing the curing temperature generally accelerates the epoxy resin curing process. However, based on the Van ‘t Hoff equation, raising the temperature in exothermic reactions, such as epoxy resin curing, reduces the equilibrium conversion rate, which subsequently decreases the cross-linking density of the resin [20]. Therefore, controlling curing parameters, particularly curing temperature and curing time, provides a means to precisely tailor the cross-linking structure within epoxy resins.

Previous research on epoxy resins has predominantly focused on enhancing elongation at break through the incorporation of various modifiers, often neglecting the critical role of the crosslinked network structure in determining mechanical properties. This oversight has led to improvements in ductility at the expense of mechanical strength. Therefore, a comprehensive understanding and precise control of the crosslinked network architecture are essential for optimizing the mechanical performance of epoxy resins. This study focuses on modulating the mechanical properties of epoxy resins by adjusting key factors that influence crosslink density, including the content of curing agents and hyperbranched polymers, along with curing conditions. The objective is to develop an optimized toughening strategy that minimizes any reduction in material strength. The findings contribute to advancing high-performance epoxy materials suited to modern engineering applications, particularly those requiring a balance of resilience and mechanical durability.

## 2. Materials and Methodology

### 2.1. Materials and Sample Preparation

#### 2.1.1. Epoxy Resin

The epoxy resin used in this study was glycidyl ether of bisphenol A, with an epoxide equivalent weight of 196 g/mol. The curing agents are mainly composed of amines (e.g., ethylenediamine, diethylenetriamine, etc.). The recommended ratio of epoxy resin to curing agent is 56:44.

#### 2.1.2. Hyperbranched Polymer

Hyperbranched epoxy resin (Hyper E102) was chosen as the toughening agent for this study, supplied by Wuhan HyperBranched Polymers Science & Technology Co., Ltd. (Wuhan, China). The synthesis pathway for E102 is detailed in the literature [18], and its fundamental properties are provided in Table 1.

#### 2.1.3. Sample Preparation

**Figure 1:**
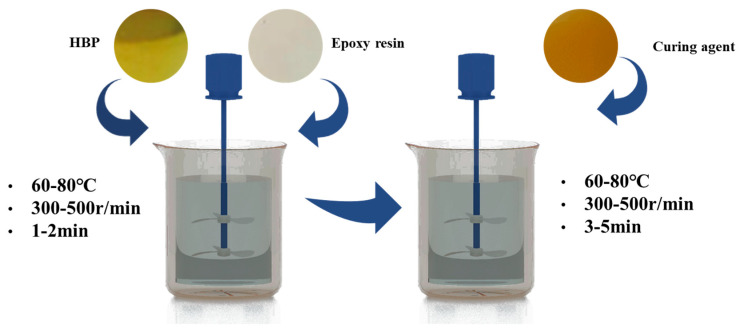
Process diagram of preparation for the epoxy resin.

The preparation method of epoxy resin significantly affects its resulting properties. To ensure a uniform blend, the epoxy resin, curing agent, and hyperbranched polymer (HBP) were preheated to 60–80 °C, as shown in Figure 1. A measured quantity of epoxy resin and HBP was then combined and stirred at 300–500 rpm for 1–2 min. Following this, the curing agent was added and mixed at the same stirring rate for an additional 3–5 min, after which the curing process was initiated.

### 2.2. Tests and Measurements

#### 2.2.1. Differential Scanning Calorimetry (DSC)

The curing behavior of the epoxy resin was scrutinized via a differential scanning calorimeter (DSC8000, PerkinElmer, Waltham, MA, USA). The samples were tested within a nitrogen flow environment, heating from 50 °C to 300 °C at varying heating rates of 5, 10, 20, and 40 K/min.

#### 2.2.2. Tensile Test

The tensile strength and elongation at break of the epoxy asphalt binders were evaluated using a computer-controlled electronic universal testing machine (EUT-5505, Senstest, Shenzhen, China) in accordance with ASTM D638 [25] at 23 °C. Dumbbell-shaped specimens were prepared, and a strain rate of 500 mm/min was applied during testing.

#### 2.2.3. Dynamic Mechanical Analysis (DMA)

Dynamic mechanical analysis (DMA) was conducted on a Discovery DMA 850 (TA Instrument, New Castle, DE, USA). Dynamic mechanical tests were carried out at a frequency of 1 Hz under a tension mode and a heating rate of 3 K/min from −30 to 100 °C.

#### 2.2.4. Thermogravimetric Analysis (TGA)

Thermogravimetric analysis (TGA) was performed within a nitrogen atmosphere by utilizing a TG209 F3 (NETZSCH, Selb, Germany). Samples, with an approximate mass of 4 mg, were subjected to heating within a nitrogen atmosphere (at a flow rate of 20 mL/min) from an initial temperature of 50 °C to a final temperature of 600 °C, with a heating rate of 10 °C/min.

#### 2.2.5. ATR-FTIR

To identify functional groups in the epoxy resin with varying curing agent and hyperbranched polymer (HBP) contents, attenuated total reflection Fourier transform infrared spectroscopy (ATR-FTIR) was performed using a Nicolet IS50 (Thermo Scientific, Waltham, MA, USA). FTIR spectra were collected in the range of 4000–400 cm^−1^ with a scanning resolution of 0.09 cm^−1^. Each sample was tested in triplicate to ensure data reliability.

## 3. Results and Discussion

### 3.1. Effect of Curing Agent and HBP Content on Epoxy Resin Properties

#### 3.1.1. Non-Isothermal Curing Kinetics

**Figure 2:**
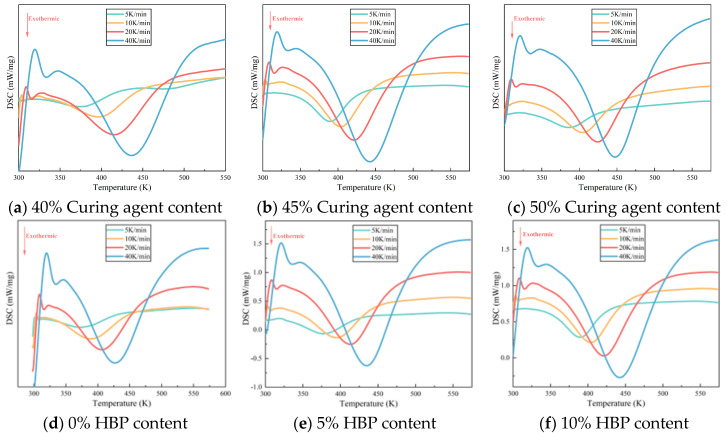
DSC curve of epoxy resin with different curing agent and HBP contents.

The curing behavior of epoxy resin is markedly influenced by variations in curing agent and hyperbranched polymer (HBP) content, which subsequently impacts its performance characteristics [14,26]. Figure 2 illustrates the differential scanning calorimetry (DSC) curves for epoxy resins with differing proportions of curing agent and HBP. As the curing process for epoxy resin is exothermic, an increase in heating rate shifts the onset, peak, and end temperatures of the exothermic peaks to higher values, resulting in a sharper exothermic profile and a reduction in curing time. This shift is primarily driven by two factors: (1) the increased heating rate augments thermal input per unit time, intensifying thermal lag and thereby shifting the reaction peak to higher temperatures, and (2) the elevated heating rate accelerates the curing reaction by increasing the reaction temperature, which shortens the curing time.

To independently assess the effects of curing agent content and hyperbranched polymer (HBP) incorporation on the properties of epoxy resins, Figure 2a–c present data from samples formulated without HBP (0 wt%), thereby isolating the influence of varying curing agent concentrations. Conversely, Figure 2d,e correspond to samples in which the curing agent content is fixed at 45 wt%, enabling evaluation of the effects associated solely with changes in HBP content. No further elaboration is required, as the following text remains consistent. The curing agent and HBP content in each sample is shown in Table 2.

Figure 2a–c demonstrate that, under a constant heating rate (such as 40 K/min), an increase in curing agent content results in a higher peak temperature on the DSC curve, indicating a requirement for extended curing time and additional heat input to achieve an equivalent degree of curing. This suggests that the activation energy barrier for the curing reaction rises with increased curing agent content. An excess of curing agent, particularly amines, can lead to the formation of alternative reaction pathways or side reactions, such as secondary amine addition and epoxy group self-polymerization. These unintended reactions may involve less reactive species or result in the formation of stable intermediates, thereby increasing the overall activation energy required for the curing process. A similar pattern is observable in Figure 2d–f, underscoring that as HBP content increases, the system’s resistance to curing also intensifies.

**Figure 3:**
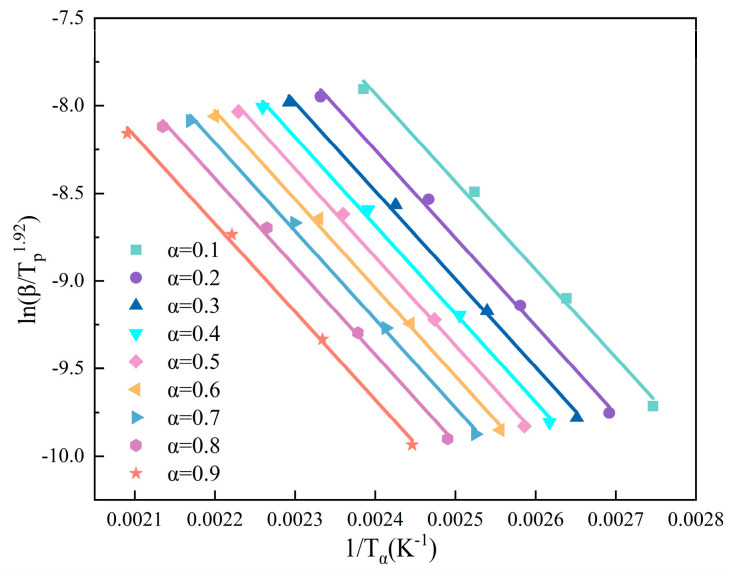
*E_a_* fitting curve based on the Starink method.

The study of epoxy curing kinetics based on the DSC method is mainly based on two basic assumptions: (1) the rate of the curing reaction is proportional to the rate of heat flow and (2) the exothermic enthalpy of the curing reaction is proportional to the degree of curing [27,28].

(1) $$\frac{d\alpha }{dt}=\frac{dH}{dt}\cdot \frac{1}{\Delta H}=k(T)f(\alpha )$$

where *f*(*α*) is the reaction model and *k*(*T*) is the rate constant expressed by the Arrhenius equation:

(2) $$k(T)=A\exp\left(-E_{a}/RT\right)$$

where A is the pre-exponential factor, *E_a_* is the activation energy (kJ/mol), and R is the gas constant, i.e., 8.314 (J/mol/K). The *E_a_* was calculated by the Starink method [9,27,28], as shown in Figure 3.

(3) $$\ln\left(\frac{\beta }{{T_{a,i}}^{1.92}}\right)=Const-1.0008\frac{E_{a}}{RT_{a,i}}$$

where *β* is the heating rate and *T*_*α*,*i*_ is the temperature at a specific value which achieved α conversion rate.

**Figure 4:**
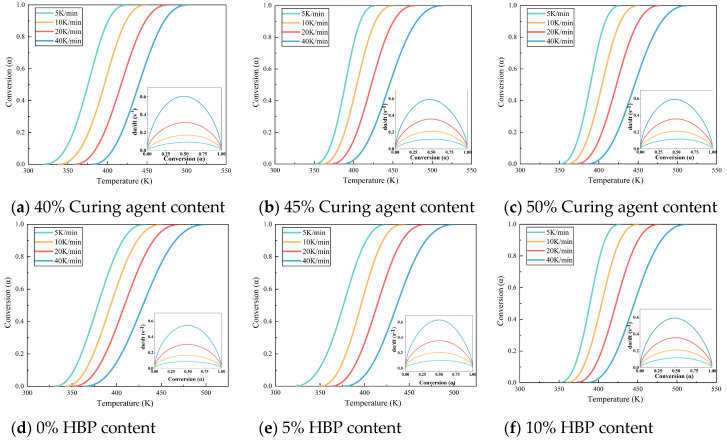
Temperature–conversion and conversion–da/dt curves of epoxy resin with different curing agent and HBP contents.

Figure 4 illustrates the temperature–conversion relationship curves for epoxy resins formulated with varying amounts of curing agent and HBP. These curves indicate that, at a constant degree of cure, the reaction rate intensifies with increasing temperature ramp rates. This trend underscores the effectiveness of elevated temperatures in accelerating the curing process, attributable to two key factors: (1) higher temperatures elevate the kinetic energy of molecular components, enabling them to more readily surpass the activation energy barrier required for the reaction, and (2) the curing process is autocatalytic, meaning that the formation of curing products actively enhances the progression of the reaction.

**Figure 5:**
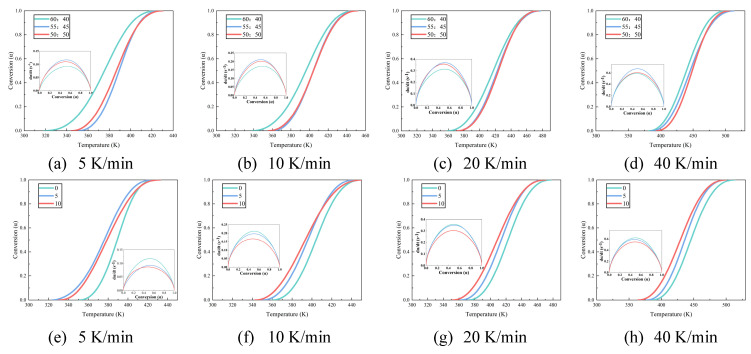
Temperature–conversion and conversion–da/dt curves of epoxy resin under different heating rates.

Figure 5 illustrates the relationship between temperature and conversion rate for epoxy resin at different heating rates. Figure 5a–d examine the influence of systematic variation in the epoxy resin to curing agent ratio on the curing kinetics while Figure 5e–h assess how graded additions of HBP modifiers alter these curing parameters. The data indicate that, when the same conversion rate is achieved, the curing rate of the epoxy resin initially increases and then decreases as the proportion of curing agent increases, which can be attributed to the intricate interplay between reactant concentration and reaction order. Specifically, the reaction order *n* between the epoxy resin and curing agent is a non-integer, which results in a complex dependency of the curing rate on reactant concentration [27]. Furthermore, the curing rate of epoxy resin decreases with increasing HBP content, likely due to the initiation of secondary reactions triggered by the HBP. These secondary reactions create competing pathways that diminish the efficiency of the primary curing reaction, thereby slowing the overall curing process.

It was shown that the autocatalytic model (Šesták–Berggren model) (Equation (4)) is a suitable choice for characterizing the intricate curing process of epoxy materials [27]:

(4) $$\frac{d\alpha }{dt}=A\exp\left(\frac{-E_{a}}{RT}\right)\alpha^{m}{\left(1-\alpha \right)}^{n}$$

where *m* is the number of reaction stages with the epoxy resin, *n* is the number of reaction stages with the curing agent, and *p* = *m*/*n* = $\alpha_{M}/\left(1-\alpha_{M}\right)$. Equation (4) can be, therefore, transformed into Equation (5):

(5) $$\ln\left[\left(\frac{d\alpha }{dt}\right)\exp\left(\frac{E_{a}}{RT}\right)\right]=\ln A+n\ln\left[\alpha^{p}(1-\alpha )\right]$$

The curing kinetics parameters, including the pre-exponential factor *A* and reaction stage *n*, can be determined by Equation (5). The reaction rate equations and kinetic parameters for different samples are listed in Table 3 and Table 4.

**Figure 6:**
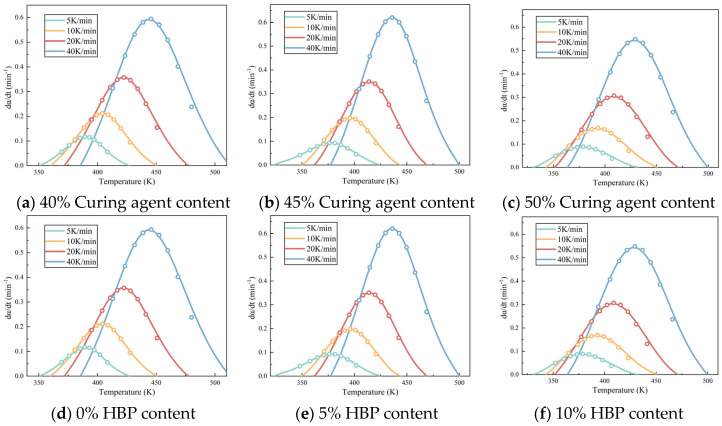
Comparison of the calculated rate (symbols) and experimental rate (lines).

A comparative analysis of the calculated values and experimental curves (Figure 6) demonstrates that the Šesták–Berggren model provides an accurate representation of the non-isothermal curing process of the epoxy system, underscoring its effectiveness in capturing the complex kinetics of epoxy resin curing. The results of Table 3 indicate that as the curing agent content increases, the activation energy of the epoxy resin system also increases. This phenomenon can be attributed to two primary factors: first, the reduction in the concentration of epoxy resin, which diminishes the collision efficiency between reactants; and second, the accumulation of unreacted epoxy resin or curing agent, which can initiate side reactions that require higher activation energies to proceed.

The reaction order parameters, *n* and *m*, which correspond to the number of reaction stages for the epoxy resin and curing agent, respectively, provide insight into the complexity of the curing process. An increase in these parameters signifies a more complex reaction mechanism. As can be seen in Table 3 and Table 4, the increase in both *n* and *m* with rising curing agent content suggests that a more intricate curing reaction mechanism is involved as the proportion of curing agent increases. Furthermore, the observed trend of the curing rate initially increasing and then decreasing with higher curing agent content, as shown in Figure 5, can be explained by the inverse relationship between the epoxy resin and curing agent proportions, with a higher curing agent content leading to a corresponding decrease in epoxy resin content.

Similarly, as shown in Table 4, the activation energy of the epoxy resin system increases with higher hyperbranched polymer (HBP) content. This increase is likely due to the side reactions between HBP and epoxy resin, which generally require higher activation energies. As HBP primarily interacts with the epoxy resin components, the number of reaction stages increases, further complicating the curing reaction mechanism as the curing agent content increases.

The curing kinetics equation allows for the calculation of the time required for epoxy resin to reach a specific conversion rate at a given temperature. Prior research [29] has established that a conversion rate of 95% or greater signifies a near-complete reaction of the epoxy resin with the curing agent. Table 5 and Table 6 present the calculated times for achieving conversion rates between 95% and 99% under curing conditions of 140 °C, 150 °C, and 160 °C, which can be calculated using Equation (4). As shown, the time to achieve a given conversion rate decreases with increasing temperature, indicating that raising the curing temperature is an effective method to shorten curing times. Moreover, under constant temperature conditions, the relationship between conversion rate and curing time is non-linear. Specifically, the time to reach a 99% conversion rate is more than double that required to achieve 98%, illustrating the substantial increase in curing time as the reaction nears completion. As a result, the time required to attain 98% conversion was selected as the primary reference for determining subsequent curing conditions in this study.

Furthermore, the time required to achieve the same conversion rate at a given temperature increases with higher curing agent content. This trend offers crucial information for optimizing curing conditions in epoxy resin systems.

#### 3.1.2. Tensile Properties

**Figure 7:**
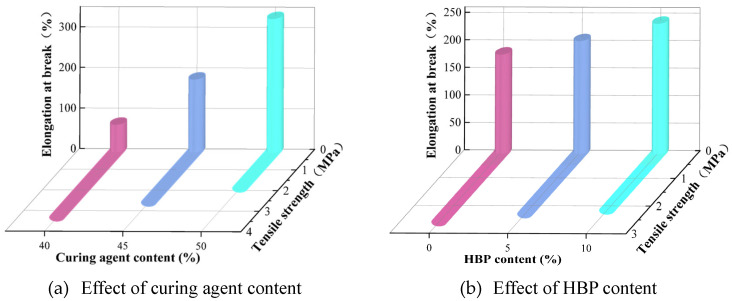
Effect of curing agent and HBP content on tensile properties of ER.

Figure 7 illustrates a clear trend in which the tensile strength of epoxy resin decreases steadily with increasing levels of curing agent and HBP, while the elongation at break correspondingly increases as the content of both components rises. Notably, the influence of curing agent and HBP on the tensile properties of epoxy resin varies in magnitude. For instance, when the curing agent content is increased from 40% to 50%, the elongation at break undergoes a substantial rise, from 68% to 330%, amounting to an increase of 385%. In comparison, raising the HBP content from 0% to 10% leads to a more modest increase in elongation at break, from 180% to 237%, a difference of 31.7%.

While adjusting the curing agent or HBP content can effectively improve the stretchability of epoxy resins, this approach is accompanied by a significant decline in tensile strength. Thus, optimizing the content of curing agent and HBP requires a balanced consideration of multiple performance metrics, as relying solely on a single property would not yield an optimal formulation.

#### 3.1.3. Thermal Stability

**Figure 8:**
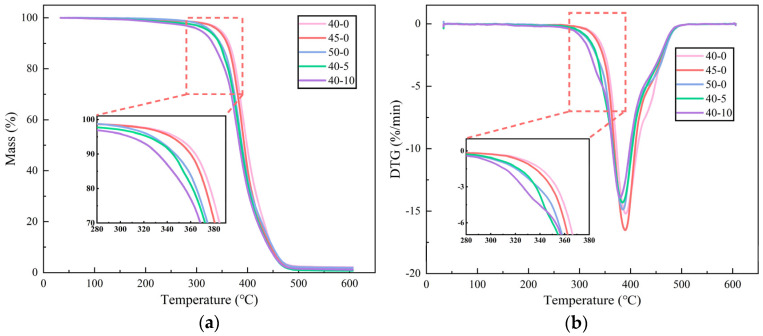
TGA (**a**) and DTG (**b**) plots of ER with different curing agent and HBP contents (the notation “40-0” specifies samples containing 40% curing agent and no HBP).

Figure 8 illustrates the thermogravimetric (TG) analysis of epoxy resin samples with varying proportions of curing agent and hyperbranched polymer (HBP). Under inert atmospheric conditions, the derivative thermogravimetric (DTG) curve exhibits a single prominent peak, corresponding to the primary thermal decomposition phase.

The epoxy resin demonstrates negligible mass loss (less than 2%) from room temperature to 300 °C, reflecting high thermal stability in this range. Beyond 300 °C, the decomposition rate accelerates significantly. It is noteworthy that increasing the curing agent and HBP content results in a progressive reduction in the onset temperature for rapid decomposition, accompanied by an increase in the decomposition rate. These observations indicate a decline in thermal stability, which is attributed to the diminished integrity of the cross-linked network structure within the epoxy resin.

#### 3.1.4. ATR-FTIR

**Figure 9:**
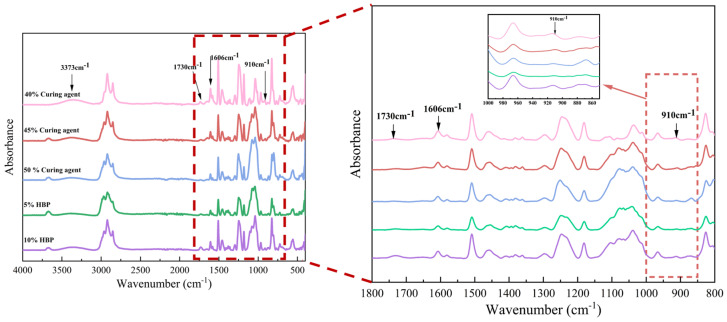
FTIR spectra of epoxy asphalt with different curing agent and HBP contents.

The impact of curing agent and hyperbranched polymer (HBP) content on the chemical composition of epoxy resins was systematically evaluated using Fourier Transform Infrared (FTIR) spectroscopy, as depicted in Figure 9. The findings demonstrate a reduction in the absorption intensity of the epoxy group (910 cm^−1^) with an increasing curing agent proportion. This decline is attributed to two factors: the decreased relative content of epoxy resin and the ring-opening reactions between epoxy groups and the curing agent, which consume these functional groups [30]. In contrast, the epoxy group absorption peak initially increases and subsequently decreases with the progressive addition of HBP. This behavior is attributed to the highly branched architecture of HBPs, which facilitates the reaction of terminal epoxy groups with the curing agent. However, excessive HBP content leads to an accumulation of unreacted epoxy groups due to incomplete reactions.

The ring-opening reaction between the epoxy groups in the resin and amino groups in the curing agent results in the formation of hydroxyl groups, as indicated by the absorption peak at 3373 cm^−1^. A slight decrease in the intensity of this peak is observed with higher curing agent proportions, potentially due to a reduction in hydroxyl group formation when excess curing agent is present. Conversely, the incorporation of HBP enhances the reaction extent, increasing hydroxyl group generation and intensifying the corresponding absorption peak.

The absorption peak associated with the C=C bonds in aromatic rings (1606 cm^−1^) remains largely unchanged, signifying that aromatic rings do not participate in the chemical reactions between the epoxy resin and curing agent. However, at an HBP content of 10%, a significant enhancement in the carbonyl group absorption peak (1730 cm^−1^) is observed. This increase might be attributed to oxidative side reactions induced by excessive HBP.

These results elucidate the intricate interactions between curing agent and HBP content in shaping the chemical composition and reaction mechanisms of epoxy resins. Such insights are critical for optimizing resin formulations to achieve tailored mechanical and chemical properties for advanced applications.

### 3.2. Optimization of Curing Conditions Based on Orthogonal Experiments

#### 3.2.1. Orthogonal Experimental Design

Recognizing that curing conditions, including curing temperature and curing time, significantly impact the curing behavior of epoxy resins, this section employs an orthogonal testing approach to examine how various preparation parameters affect the mechanical properties of the resin. Four key factors were selected for analysis: high curing temperature, high-temperature curing time, low curing temperature, and low-temperature curing time. Based on the peak temperature range observed in the DSC curves in Section 2.2.1., the high curing temperature was set between 140 °C and 160 °C, and the curing time was set between 1 and 2 h, as indicated by the results in Table 5 and Table 6. Following the recommendations from the manufacturer, the low curing temperature was selected to be between 50 °C and 70 °C, with a curing time of 2 to 4 days. Each of these factors was examined at three distinct levels, as detailed in the accompanying Table 7.

#### 3.2.2. Curing Conditions Optimization

Epoxy resin specimens were prepared according to the ASTM D638-2022 [25] (Type IV) standard, with an epoxy resin-to-curing agent ratio of 56:44 across various factor levels. Direct tensile tests were then conducted at 23 °C, and the tensile strength and elongation at break obtained from these tests were analyzed as outputs of the orthogonal experiment.

An intuitive analysis, also referred to as analysis of variance (ANOVA), was employed to quantify the effect of each factor by calculating the R-value, which represents the extreme variance for each factor. This approach allows for the identification of the optimal level for each factor and, ultimately, the determination of the optimal parameter combination. In this context, *k* denotes the mean value of each parameter at a specific level, with the magnitude of extreme variance indicating the sensitivity of experimental results to changes in factor levels. Table 8 and Table 9 presents the visual analysis results, with tensile strength and elongation at break serving as evaluation metrics.

Table 10 indicates that the factors affecting tensile strength are ranked, in descending order of influence, as follows: high curing temperature, high-temperature curing time, low curing temperature, and low-temperature curing time. Conversely, the factors influencing elongation at break are ranked in order of impact as low curing temperature, low-temperature curing time, high-temperature curing time, and high curing temperature (Table 11).

While the intuitive analysis method provides a ranking of factor influence, it does not offer a precise quantitative assessment of significance of each factor. Therefore, analysis of variance (ANOVA) was employed to test the statistical significance of the effects of each factor.

**Figure 10:**
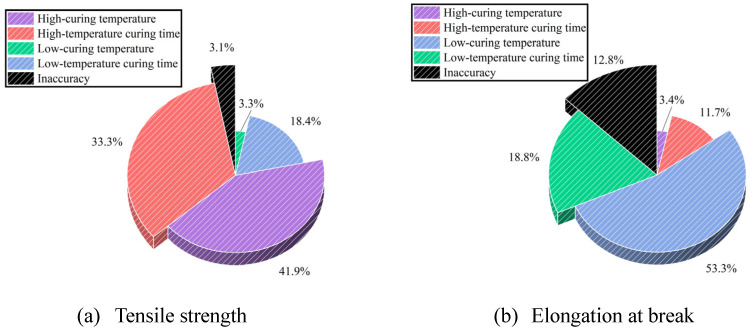
Factors affecting tensile strength and elongation at break.

A sector diagram (Figure 10) was constructed to illustrate the contribution of each factor, facilitating a clear analysis of their effects. The diagram indicates that high curing temperature is the most influential factor for tensile strength, followed by high-temperature curing time, with these two factors collectively accounting for over 70% of the impact on tensile strength. For elongation at break, low curing temperature emerges as the primary contributor, influencing more than 50% of the property outcome.

To enhance toughness with minimal compromise to tensile strength, this study established fixed curing parameters based on previous extreme difference analysis. The high curing temperature was set to 140 °C, high-temperature curing time to 1.5 h, and low-temperature curing time to 2 days. Only the low curing temperature was varied, allowing for dynamic control over the epoxy resin’s toughness.

### 3.3. Toughness Enhancement of Epoxy Resins Based on Crosslink Density Regulation

#### 3.3.1. Crosslink Density Regulation

This study seeks to achieve precise control over the crosslink density of epoxy resin by adjusting the curing agent proportion, low curing temperature, and hyperbranched polymer (HBP) content, utilizing response surface methodology as the primary analytical approach. The primary objective is to enhance the toughness of the epoxy resin efficiently while minimizing any potential loss in mechanical strength. To facilitate this, a multi-objective optimization function *Z* is developed (Equation (6)). The response surface model incorporates curing agent content (40–50%), HBP content (0–10%), and low curing temperature (50–70 °C) as independent variables. The response outputs include the multi-objective optimization function *Z* and the crosslink density ($\nu_{e}$, mol/m^3^) of the epoxy resin, with the latter calculated from the storage modulus in the rubbery plateau region based on rubber elasticity theory (Equation (7)) [31]:

(6) $$Z=w_{1}*\frac{E_{b}}{E_{\mathrm{initial}}}-w_{2}*\frac{T_{loss}}{T_{initial}}$$

where $E_{b}$ is the elongation at break, $E_{\mathrm{initial}}$ is the initial value of elongation at break, $T_{loss}$ is the loss of strength, $T_{initial}$ is the initial value of strength, and $w_{1}$ and $w_{2}$ are the scaling factors, which were both set to 0.5 in this study.

(7) $$\nu_{e}=\frac{E^{\prime }}{3RT}$$

where $E^{\prime }$ denotes the storage modulus at the point where tanδ (equal to $E^{\prime }/E^{\prime \prime }$) reaches its maximum, *R* is the universal gas constant (8.314 J/(K mol)), and *T* is the absolute temperature (K), calculated as *T_g_* + 30 or 40 K.

Based on the preceding analysis, the Box–Behnken design (BBD) within the response surface methodology framework was employed to structure the experimental design. To enable the calculation of pure error, five center points were included, with each factor’s coding and testing levels provided in Table 12.

#### 3.3.2. Influence of Parameters on the Crosslink Density of Epoxy Resin

Experimental simulations were conducted according to the test combinations specified in the Box–Behnken design (BBD), utilizing the coded factor levels detailed in the table. The resulting data, including the epoxy crosslink density (*Y*_1_) and optimization function value (*Y*_2_), are summarized in the following Table 13.

After evaluating various fitting models, the linear model was chosen based on its *p*-values and *R*^2^ values, as it effectively represents the relationship between crosslink density and the factors *X*_1_, *X*_2_, and *X*_3_. Stepwise regression and an F-test were then applied, with statistical outcomes reported in Table 14.

A model *p*-value of less than 0.0001 confirms its high level of statistical significance, while a lack-of-fit *p*-value of 0.2875 indicates that the lack of fit is not statistically significant relative to pure error, underscoring the model’s robustness and strong correlation. The model’s coefficient of determination, *R*^2^, at 0.981, further demonstrates its predictive accuracy. Furthermore, the *p*-values for factors *X*_1_, *X*_2_, and *X*_3_ were each below 0.05, indicating that low curing temperature, curing agent content, and HBP content significantly influence the crosslink density of the epoxy resin. Importantly, the interaction between low curing temperature and HBP content also significantly affects crosslink density.

**Figure 11:**
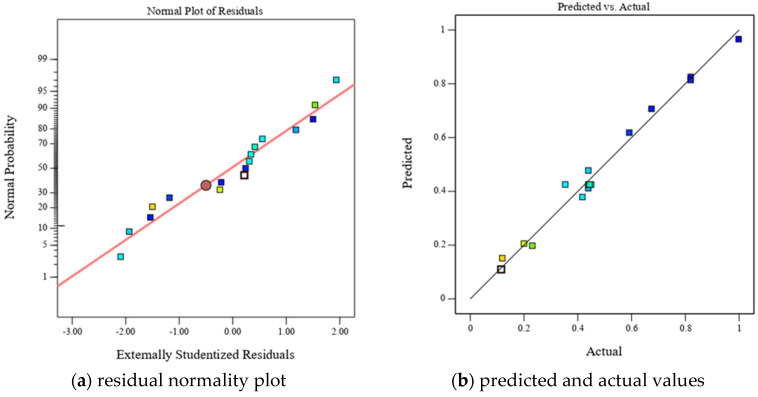
Reliability evaluation of crosslink density prediction model.

Figure 11 illustrates the residual normality plot and the relationship between predicted and observed values for epoxy resin crosslink density. The close alignment of data points along the diagonal suggests a high degree of normality and reproducibility in the data. To further assess the influence of each factor on crosslink density, three-dimensional response surface plots were constructed.

**Figure 12:**
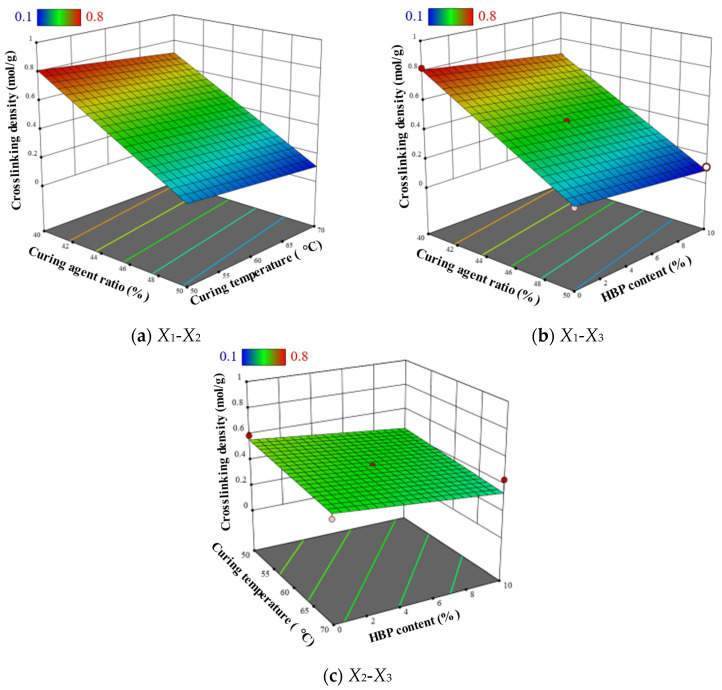
Response surface plots of crosslink density prediction model.

Given the linear model, the response surfaces are represented as planar (Figure 12). The analysis reveals that an increase in curing agent content from 40% to 50% substantially reduces crosslink density from 0.82 mol/g to 0.12 mol/g. Exceeding the ideal epoxy-to-amine molar ratio breaks the balance required for full network formation, leaving unreacted functional groups and promoting shorter, less-connected chains that lower effective crosslink density. Additionally, an excess of amine curing agent increases the likelihood of side reactions, which compete with the desired epoxy–amine addition and consume epoxy groups, diminishing the number of sites available for true crosslinking. Likewise, elevating the low curing temperature from 50 °C to 70 °C decreases crosslink density from 0.23 mol/g to 0.12 mol/g. Additionally, increasing the HBP content from 0% to 10% results in a reduction in crosslink density from 0.2 mol/g to 0.11 mol/g. This is due to the highly branched spatial structure of HBP, which leads to an increase in the free volume of ER. These results indicate that curing agent content has a pronounced effect on crosslink density, while low curing temperature and HBP content exert relatively minor effects.

**Figure 13:**
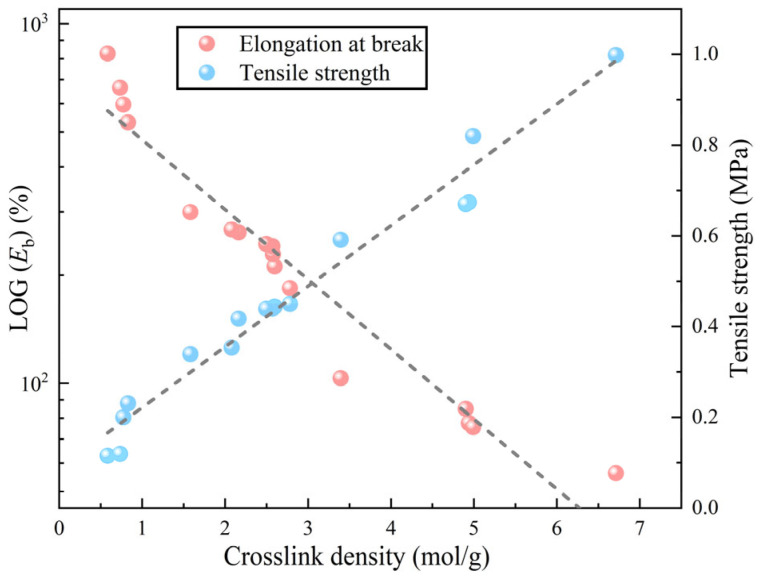
Linear relationship between crosslink density and mechanical parameters.

The crosslink density was linearly fitted against tensile strength and elongation at break in logarithmic coordinates (Figure 13). The results demonstrate a strong positive correlation between crosslink density and tensile strength, as well as a strong negative correlation between crosslink density and elongation at break. These findings suggest that controlling crosslink density is an effective strategy for tuning the mechanical properties of epoxy resins. However, they also imply that a decrease in strength is almost inevitable when attempting to improve toughness. Therefore, achieving enhanced toughness with minimal strength reduction becomes a key objective for optimizing epoxy resin performance.

#### 3.3.3. Influence of Parameters on the Toughness of Epoxy Resin

After evaluating various fitting models through *p*-values and R^2^ values, a quadratic model was identified as the most suitable for capturing the relationship between the multi-objective toughness optimization index *Z* and factors *X*_1_, *X*_2_, and *X*_3_. An F-test, applied via stepwise regression, yielded the statistical results presented in Table 15. The model’s *p*-value of 0.001 signifies a high degree of statistical significance, while the lack-of-fit *p*-value of 0.1407 indicates no significant deviation from pure error, thus affirming the model’s robustness and high correlation. With a coefficient of determination R^2^ = 0.9483, the model demonstrates excellent predictive accuracy. Additionally, the quadratic terms X_1_^2^, X_2_^2^, and X_3_^2^ all have *p*-values below 0.05, underscoring that the quadratic effects of these factors significantly influence the *Z* index, crucial for optimizing epoxy resin toughness.

**Figure 14:**
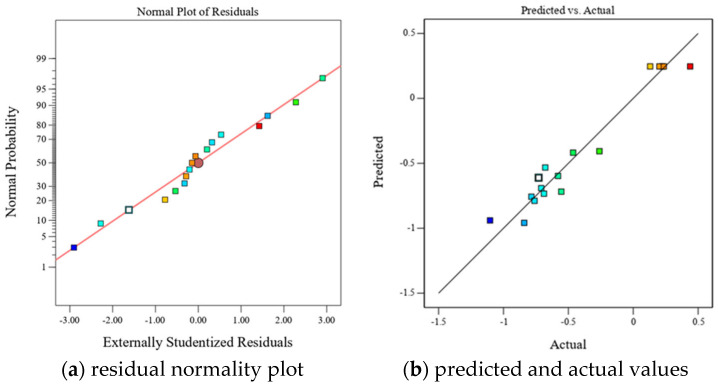
Reliability evaluation of optimization function *Z* prediction model.

**Figure 15:**
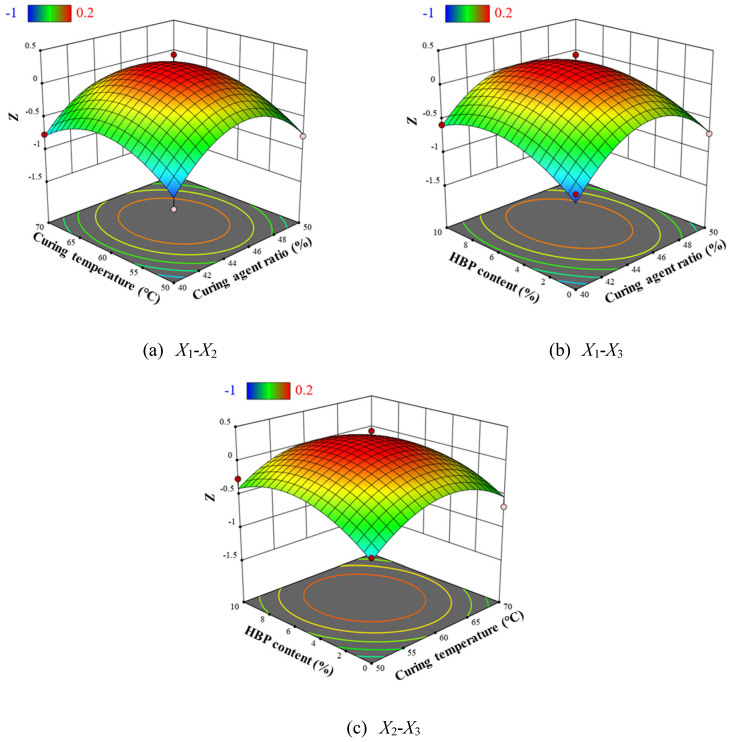
Response surface plots of optimization function *Z* prediction model.

Figure 14 presents the residual normality plot and the correlation between predicted and actual values of *Z* index. The data distributions are both close to the diagonal, illustrating compliance with normality requirements and good data reproducibility. The effect of each factor on the *Z* index of epoxy resin was further analyzed by establishing three-dimensional response surface plots (As shown in Figure 15). The Z index tends to increase and then decrease with the increase of curing agent content and HBP content and low curing temperature, which indicates that excessive reduction of crosslink density is not a reasonable means of toughening. From the contour density, the three factors have similar influence on the Z index of epoxy resin. To obtain the most ideal toughness optimization scheme, we extracted the maximum value of Z index, under which the curing agent content was 45.21%, the low curing temperature was 60.45 °C, and the HBP content was 5.77%. Under this condition, the test and predicted values of mechanical properties of epoxy resins are shown in Table 16.

## 4. Conclusions

This article proposed an efficient toughness modification strategy based on multi-objective optimization to achieve a balance between toughness enhancement and strength loss by modulating the crosslinking density of epoxy resin. The specific conclusions are as follows:

(1) The curing agent and hyperbranched polymer (HBP) content exert a significant influence on the epoxy resin curing reaction. Specifically, the curing rate initially increases with the proportion of curing agent, but subsequently decreases beyond a certain concentration. In contrast, the curing rate decreases as the HBP content increases. These trends are attributed to variations in reactant concentration and the occurrence of side reactions, which impact the overall curing process.
(2) The stretchability of epoxy resins increases with higher curing agent and HBP content, which is accompanied by a corresponding reduction in mechanical strength and thermal stability. Notably, the curing agent content exerts a more pronounced influence on the mechanical properties of ER compared to HBP content.
(3) High curing temperatures exert the most significant influence on the strength of epoxy, trailed by extended curing times. In contrast, low curing temperatures have the predominant effect on the toughness of epoxy.
(4) The crosslink density of epoxy resin manifested a continuous decline with the augmentation of curing agent, HBP content, and low curing temperature, and the curing agent content exerted the most substantial influence. The crosslink density exhibits a highly linear correlation with strength and elongation at break, substantiating that the manipulation of crosslink density represents an efficacious strategy for modulating the mechanical properties of ER.
(5) The *Z* index exhibited a trend of initial increment followed by subsequent decrement with the augmentation of curing agent and HBP content as well as low curing temperature, implying that an excessive reduction in crosslink density is not justifiable. The present study furnished the optimal solution: a curing agent content of 45.21%, a low curing temperature of 60.45 °C, and an HBP content of 5.77%.

## Figures and Tables

**Table 1:** Basic indexes of E102.

Properties	Units	Test Results	Test Methods
Appearance	-	Yellow transparent liquid	Visual Observation
Epoxy value	mol/100 g	0.15 ± 0.05	GB/T1677 [21]
Solid content	%	≥95	GB/T-2793 [22]
VOCs	%	≤5.0	GB/T-23986.2 [23]
Molecular weight	g/mol	3200–3600	GB/T-31816 [24]

**Table 2:** The curing agent and HBP content in each sample.

Sample	Curing Agent Content	HBP Content
1	40	0
2	45	0
3	50	0
4	45	0
5	45	5
6	45	10

**Table 3:** Curing kinetics parameters of epoxy resin with different curing agent contents.

Curing Agent Content	Reaction Kinetic Equation	*E_a_ (J/mol)*	*n*	*m*	*lnA* *(min^−1^)*
40	*dα/dt = 3.70 × 10^4^exp(−37,446/RT)α^0.247^(1 − α)^0.998^*	37,446	0.998	0.247	10.514
45	*dα/dt = 1.29 × 10^5^exp(−41,776/RT)α^327^(1 − α)^1.037^*	41,776	1.037	0.302	11.770
50	*dα/dt = 2.46 × 10^5^exp(−43,942/RT)α^372^(1 − α)^1.089^*	43,942	1.089	0.327	12.411

**Table 4:** Curing kinetics parameters of epoxy resin with different HBP contents.

HBP Content	Reaction Kinetic Equation	*E_a_ (J/mol)*	*n*	*m*	*lnA* *(min^−1^)*
0	*dα/dt = 1.29 × 10^5^exp(−41,776/RT)α^0.327^(1 − α)^1.037^*	41,776	1.037	0.302	11.770
5	*dα/dt = 2.67 × 10^5^exp(−44,530/RT)α^0.235^(1 − α)^1.095^*	44,530	1.095	0.235	12.496
10	*dα/dt = 1.89 × 10^6^exp(−49,761/RT)α^0.160^(1 − α)^1.331^*	49,761	1.331	0.160	14.450

**Table 5:** Time required for ER to achieve conversion under different curing agent content conditions.

Curing Temperature	α	Curing Time (min)		
		40% ER	45% ER	50% ER
140 °C	95%	28.0	32.0	37.0
	96%	35.3	40.7	47.4
	97%	47.4	55.2	65.3
	98%	71.7	84.6	102.2
	99%	144.2	174.7	218.8
150 °C	95%	21.7	24.0	27.3
	96%	27.3	30.5	35.1
	97%	36.7	41.4	48.3
	98%	55.4	63.5	75.5
	99%	111.5	131.1	161.7
160 °C	95%	17.0	18.3	20.5
	96%	21.4	23.2	26.3
	97%	28.7	31.5	36.2
	98%	43.3	48.2	56.6
	99%	87.2	99.7	121.2

**Table 6:** Time required for ER to achieve conversion under different HBP content conditions.

Curing Temperature	α	Curing Time(min)		
		0% HBP	5% HBP	10% HBP
140 °C	95%	32.0	40.9	53.5
	96%	40.7	52.6	72.7
	97%	55.2	72.6	107.5
	98%	84.6	114.1	186.1
	99%	174.7	245.7	472.1
150 °C	95%	24.0	30.1	38.0
	96%	30.5	38.7	51.6
	97%	41.4	53.5	76.4
	98%	63.5	84.0	132.1
	99%	131.1	180.9	335.2
160 °C	95%	18.3	22.5	27.4
	96%	23.2	28.9	37.2
	97%	31.5	39.9	55.1
	98%	48.2	62.7	95.3
	99%	99.7	135.0	241.8

**Table 7:** Four-factor, three-level design of orthogonal experiments.

High Curing Temperature (°C)	High-Temperature Curing Time (h)	Low Curing Temperature (°C)	Low-Temperature Curing Time (d)
140	1	50	2
150	1.5	60	3
160	2	70	4

**Table 8:** Intuitive analysis using tensile strength as an evaluation index.

	High Curing Temperature (°C)	High-Temperature Curing Time (h)	Low curing Temperature (°C)	Low-Temperature Curing Time (d)	Tensile Strength (MPa)
1	140 (1)	1 (1)	50 (1)	2 (1)	5.25
2	140 (1)	1.5 (2)	70 (3)	3 (2)	3.38
3	140 (1)	2 (3)	60 (2)	4 (3)	3.52
4	150 (2)	1 (1)	70 (3)	4 (3)	3.34
5	150 (2)	1.5 (2)	60 (2)	2 (1)	3.07
6	150 (2)	2 (3)	50 (1)	3 (2)	3.00
7	160 (3)	1 (1)	60 (2)	3 (2)	3.02
8	160 (3)	1.5 (2)	50 (1)	4 (3)	3.08
9	160 (3)	2 (3)	70 (3)	2 (1)	1.92
k_1_	4.05	3.87	3.78	3.41	
k_2_	3.14	3.18	3.20	3.13	
k_3_	2.68	2.82	2.88	3.31	
R	1.37	1.05	0.57	0.28	

**Table 9:** Intuitive analysis using elongation at break as an evaluation index.

	High Curing Temperature (°C)	High-Temperature Curing Time (h)	Low Curing Temperature (°C)	Low-Temperature Curing Time (d)	Elongation at Break (%)
1	140 (1)	1 (1)	50 (1)	2 (1)	188.00
2	140 (1)	1.5 (2)	70 (3)	3 (2)	210.67
3	140 (1)	2 (3)	60 (2)	4 (3)	124.00
4	150 (2)	1 (1)	70 (3)	4 (3)	205.33
5	150 (2)	1.5 (2)	60 (2)	2 (1)	188.00
6	150 (2)	2 (3)	50 (1)	3 (2)	142.67
7	160 (3)	1 (1)	60 (2)	3 (2)	156.00
8	160 (3)	1.5 (2)	50 (1)	4 (3)	182.67
9	160 (3)	2 (3)	70 (3)	2 (1)	229.33
k_1_	174.22	183.11	171.11	201.78	
k_2_	178.67	193.78	156.00	169.8	
k_3_	189.33	165.33	215.11	170.7	
R	15.11	28.44	59.11	32.00	

**Table 10:** Analysis of variance (ANOVA) for tensile strength index.

Source of Variance	Sum of Squared Deviations	Freedom Degrees	Mean Square	F	*p*	Significance	Contribution
High curing temperature	12.37	2	6.19	121.82	<0.001	YES	41.9
High-temperature curing time	9.81	2	4.91	96.63	<0.001	YES	33.3
Low curing temperature	5.43	2	2.72	53.47	<0.001	YES	18.4
Low-temperature curing time	0.97	2	0.49	9.58	<0.001	YES	3.3
*Se* (Inaccuracy)	0.91	18	0.05	-	-	-	3.1

**Table 11:** Analysis of variance (ANOVA) for elongation at break index.

Source of Variance	Sum of Squared Deviations	Freedom Degrees	Mean Square	F	*p*	Significance	Contribution
High curing temperature	1085.63	2	542.82	2.39	0.12	NO	3.4
High-temperature curing time	3716.74	2	1858.37	8.19	<0.001	YES	11.7
Low curing temperature	16975.4	2	8487.70	37.40	<0.001	YES	53.3
Low-temperature curing time	5978.07	2	2989.04	13.17	<0.001	YES	18.8
*Se* (Inaccuracy)	4085.33	18	226.96				12.8

**Table 12:** Parameter codes and test levels.

Parameters	−1	0	1
Curing agent content X_1_ (%)	40	45	50
Low curing temperature X_2_ (°C)	50	60	70
HBP content X_3_ (%)	0	5	10

**Table 13:** BBD test results.

	Parameter Codes			Experimental Value	
	X_1_ (%)	X_2_ (°C)	X_3_ (%)	Y_1_ (mol/g)	Y_2_
1	50	50	5	0.23	−0.8
2	50	60	10	0.12	−0.7
3	50	70	5	0.12	−0.6
4	50	60	0	0.20	−0.7
5	45	60	5	0.44	0.4
6	45	50	10	0.34	−0.3
7	45	60	5	0.44	0.2
8	45	60	5	0.45	0.2
9	45	70	0	0.42	−0.7
10	40	70	5	0.67	−0.8
11	40	50	5	1.00	−1.1
12	40	60	10	0.67	−0.6
13	40	60	0	0.82	−0.8
14	45	60	5	0.35	0.1
15	45	50	0	0.59	−0.7
16	45	60	5	0.44	0.2
17	45	70	10	0.44	−0.5

**Table 14:** ANOVA results of the fitted model for crosslink density.

Variables	Sum of Squares	Degrees of Freedom	Mean Square	F-Value	*p*-Value	Significance
Model	0.7664	3	0.2555	73.49	< 0.0001	YES
X_1_	0.7183	1	0.7183	206.64	< 0.0001	YES
X_2_	0.0210	1	0.0210	6.05	0.0287	YES
X_3_	0.0271	1	0.0271	7.78	0.0153	YES
Residual	0.0452	13	0.0035			-
Lack of fit	0.0386	9	0.0043	2.62	0.1839	NO
Pure error	0.0066	4	0.0016			
Total regression	0.8116	16				

Note: R^2^ = 0.9443, Adj-R^2^ = 0.9315, Pred-R^2^ = 0.8947, C.V. = 13.12%, S/N = 25.0223.

**Table 15:** ANOVA results of the fitted model for multi-objective toughness optimization index Z.

Variables	Sum of Squares	Degrees of Freedom	Mean Square	F-Value	*p*-Value	Significance
Model	3.37	9	0.3741	14.28	0.001	YES
X_1_	0.0321	1	0.0321	1.23	0.3047	NO
X_2_	0.0181	1	0.0181	0.6907	0.4333	NO
X_3_	0.0975	1	0.0975	3.72	0.0950	NO
X_1_X_2_	0.0032	1	0.0032	0.1208	0.7384	NO
X_1_X_3_	0.0196	1	0.0196	0.7498	0.4152	NO
X_2_X_3_	0.0113	1	0.0113	0.4309	0.5325	NO
X_1_^2^	1.62	1	1.62	61.67	0.0001	YES
X_2_^2^	0.7698	1	0.7698	29.37	0.0010	YES
X_3_^2^	0.4898	1	0.4898	18.69	0.0035	YES
Residual	0.1834	7	0.0262			-
Lack of fit	0.1304	3	0.0435	3.28	0.1407	NO
Pure error	0.0530	4	0.0133			
Total regression	3.55	16				

Note: R^2^ = 0.9483, Adj-R^2^ = 0.8819, Pred-R^2^ = 0.3890, C.V. = 9.77%, S/N = 9.701.

**Table 16:** Comparison of test and predicted mechanical properties of ER under optimized conditions.

	Tensile Strength (MPa)	Elongation at Break (%)
Test value	2.52	277
Predicted value	2.48	282

## Data Availability

The original contributions presented in this study are included in the article. Further inquiries can be directed to the corresponding author.
